# Myeloid-derived suppressor cells and their subsets serve as potential biomarkers for the progression of brucellosis

**DOI:** 10.3389/fmicb.2026.1730300

**Published:** 2026-02-05

**Authors:** Juan Shi, Yibeibaihan Maimaiti, Xinxin Qi, Na Chen, Huidong Shi, Jianbing Ding, Yuejie Zhu, Fengbo Zhang

**Affiliations:** 1 Clinical Laboratory Center, The First Affiliated Hospital of Xinjiang Medical University, Urumqi, China; 2 Department of Medical Laboratory, Hospital of Xinjiang Production and Construction Corps/Second Affiliated Hospital of Shihezi University School of Medicine, Urumqi, China; 3 Post-doctoral Research station of the Clinical Medicine, The First Affiliated Hospital of Xinjiang Medical University, Urumqi, China; 4 Reproductive Medicine Center, The First Affiliated Hospital of Xinjiang Medical University, Urumqi, China

**Keywords:** *Brucella*, immunosuppression, MDSC, PD-L1, TLR4

## Abstract

**Background:**

Brucellosis remains the most prevalent zoonotic disease globally and can cause chronic persistent infection, which in turn results in prolonged recovery challenges. Myeloid-derived suppressor cells (MDSCs) are pathologically activated neutrophils and monocytes with strong immunosuppressive activity. Toll-like receptor 4 (TLR4) can initiate the body’s inflammatory response, leading to an inflammatory cytokine storm. Programmed death ligand 1 (PD-L1) modulates the strength and duration of the immune response, diminishing the immune system’s ability to eliminate pathogens and subsequently affecting disease progression and prognosis. However, the clinical significance of TLR4^+^MDSC and PD-L1^+^MDSC in *Brucella* infection remains unclear.

**Methods:**

A total of 88 patients with acute brucellosis infection (ABI), 66 patients with chronic brucellosis infection (CBI), and 82 healthy controls (HC) subjects were enrolled. Flow cytometry was used to detect TLR4^+^MDSCs and PD-L1^+^MDSCs of patients. ELISA was used to detect ALT, AST, Arg1 and iNOS in the patient’s serum. We characterized a mouse model of *Brucella*, and determined the effects of TLR4^+^MDSCs and PD-L1^+^MDSCs in this model.

**Results:**

Our study found that the frequency of MDSC in CBI group was significantly elevated. The levels of Arg1 and iNOS were also positively correlated with the levels of TLR4^+^MDSCs and PD-L1^+^MDSCs. The levels of AST and ALT had elevated may reflect liver function. In addition, we also found that the number of TLR4^+^MDSCs and PD-L1^+^MDSCs increased in model mice with chronic brucellosis.

**Conclusion:**

These findings expand the current understanding of persistent Brucella infection, and highlight that TLR4^+^ and PD-L1^+^ MDSCs hold potential as candidate biomarkers for assessing the severity and progression of brucellosis.

## 1 Introduction

*Brucella* is a Gram-negative bacterium responsible for brucellosis, a prevalent zoonotic disease globally (About et al., 2023). Conservative estimates indicate that the annual incidence rate of brucellosis is approximately 2.1 million cases with reported instances of human and animal infections in over 170 countries (Laine et al., 2023). In China, the average annual incidence rate of brucellosis is 3 cases per 100,000 people, reflecting an average annual increase of 31.8% (Tao et al., 2021). From 2004 to 2021, the incidence of human brucellosis in China has shown a sustained upward trend, with the predominant transmission route being direct contact with infected livestock, such as sheep, goats and cattle (Tian et al., 2024). Humans can contract *Brucella* through the consumption of raw dairy products and meat contaminated with this pathogen, or through direct contact with infected animal, particularly via broken mucosal wounds (Moreno, 2014). *Brucella* has the capacity to infect any organ or system within the human body, initially manifesting as mild flu-like symptoms, including fever, excessive sweating, discomfort, myalgia, arthralgia, loss of appetite, and weight loss (Cerqueira et al., 2018). If left untreated, the condition can progress from the acute to the chronic stage, leading to multiple complications across various systems. Among these, bone and joint involvement is the most prevalent complication, with a reported prevalence rate ranging from approximately 2–77%, typically presenting as spondylitis, sacroiliitis, and peripheral arthritis. Additionally, hepatosplenomegaly is observed in about 50% of patients with *Brucella melitensis*. The disease is characterized by a prolonged course and frequent recurrences, significantly impacting patients’ quality of life (Jin et al., 2023; Willems et al., 2022).

Myeloid-Derived Suppressor Cells (MDSCs) represent a highly heterogeneous subpopulation of myeloid cells characterized by their potent inhibitory effects on immune responses, particularly those mediated by natural killer (NK) cells, T cells, and B cells. Research has demonstrated the presence of MDSCs in the peripheral blood of patients suffering from various diseases (Cassetta et al., 2020). In humans, MDSCs are typically identified by the markers CD11b^+^CD33^+^HLA-DR^−^. In murine models, the MDSC phenotype is generally defined by the expression of CD11b^+^Gr1^+^ of MDSCs (Veglia et al., 2021). The immunosuppressive function of MDSCs on T-cell functions is predominantly mediated by inducible nitric oxide synthase (iNOS) and arginase 1(Arg1) (Aintablian et al., 2023; Draghiciu et al., 2015). In the hypoxic tumor microenvironment, the expression levels of Arg1 and iNOS are significantly upregulated (Kumar et al., 2016).

Toll-like receptor 4 (TLR4) is a type of Pattern Recognition Receptor (PRR) that rapidly recognizes pathogen-associated molecular patterns (PAMPs) during innate immune responses and plays a crucial role in the pathogenesis of both acute and chronic inflammation (Zhang et al., 2021; Zamyatina and Heine, 2020). In the context of *Brucella* infection, TLRs interact with MyD88 to initiate cellular signal transduction, ultimately activating the transcription factor NF-κB and regulating the expression of various inflammation-related genes (Heine and Zamyatina, 2022). The number of MDSCs in the peripheral blood of patients with chronic brucellosis is increased, and levels of Th2 cytokines, such as IL-6 and IL-10, as well as Arg1, are significantly elevated (Hou et al., 2024). This study aims to further investigate the association between the presence of TLR4^+^MDSCs in acute and chronic *Brucella* infections and their associated inflammatory changes.

Programmed death ligand 1 (PD-L1) is an inhibitory ligand that binds to programmed death 1(PD-1), activating the co-inhibitory pathway of T cells to maintain self-tolerance, thereby functioning as an immune checkpoint under physiological conditions. Immune checkpoints are a series of regulatory mechanisms in the immune system, typically employed to maintain the balance of the immune response. However, under certain pathological conditions, such as tumor development, immune checkpoints can inhibit the immune system’s attack on tumor cells, enabling these cells to evade immune surveillance. In the MDSCs of tumor-bearing mice, PD-L1 is highly expressed within the tumor microenvironment, promoting tumor progression (Lu et al., 2016). Additionally, studies have indicated that the gene expression of PD-L1 is upregulated in the whole blood of patients with brucellosis (Pellegrini et al., 2025). This study aims to investigate the presence of PD-L1^+^MDSC in the progression of acute and chronic brucellosis and to elucidate its role in disease progression.

The current literatures on the research of the expression of TLR4 and PD-1 on MDSCs in brucellosis patients at different stages are rare or unreported. In order to further clarify the role of potential biomarkers for assessing the severity and progression of brucellosis, we explored the TLR4^+^MDSCs and PD-L1^+^MDSCs in patients and mouse model with brucellosis. Establishing a robust theoretical foundation for the subsequent development of clinical treatment strategies.

## 2 Methods

### 2.1 Subjects

From January 2024 to April 2025, we collected data from 88 with ABI patients, 66 with CBI patients, and 82 with HC subjects from the First Affiliated Hospital of Xinjiang Medical University. All patients receive antibiotic treatment based on their specific conditions. The diagnostic and clinical staging criteria for brucellosis will adhere to the guidelines outlined in the ‘Diagnostic Criteria for Brucellosis WS 269-2019,’ issued by the National Health Commission of the People’s Republic of China in 2019. This study has received approval from the Ethics Committee of the First Affiliated Hospital of Xinjiang Medical University, with the ethics number K202401-18.

### 2.2 Inclusion and exclusion criteria

Patients who met the diagnostic criteria outlined in the brucellosis guidelines were included in the study. The staging criteria were defined as follows: a disease duration of less than 3 months was classified as the acute phase, three to 6 months as the subacute phase, and more than 6 months as the chronic phase. Patients were excluded from inclusion based on the following criteria: (1) patients with other infectious diseases, such as typhoid fever, paratyphoid fever, and tuberculosis; (2) patients with severe diseases affecting the respiratory, cardiovascular, cerebrovascular, or endocrine systems; (3) patients who have experienced rheumatic immune diseases within the past 5 years, as well as those using immunosuppressants or hormones; (4) patients with underlying diseases or tumors.

### 2.3 MDSCs flow cytometry

Peripheral blood mononuclear cells (PBMCs) were isolated using Ficoll density gradient centrifugation. The separated PBMCs were incubated with fluorochrome-conjugated monoclonal antibodies at 4 °C for 30 min (Monaco et al., 2019). Myeloid-derived suppressor cells (MDSCs) were labeled with antibodies against CD45 (Biolegend, USA), HLA-DR (Biolegend, USA), CD33 (BD Biosciences, USA), and CD11b (Biolegend, USA). Additionally, PD-L1-expressing cells within MDSCs were defined as PD-L1^+^MDSCs, while TLR4-expressing cells within MDSCs were designated as TLR4^+^MDSCs. Following two washes with phosphate-buffered saline (PBS), the samples were resuspended to form a single-cell suspension, which was subsequently prepared for flow cytometric analysis. Data acquisition was performed using a BD FACSLyric™ flow cytometer, and data analysis was conducted with FlowJo software.

### 2.4 ELISA detection

Serum samples were collected from patients with brucellosis, and the serum levels of alanine transaminase (ALT) and aspartate transaminase (AST) were measured using an automatic biochemical analyzer (Roche, Germany) to assess the degree of liver injury. Furthermore, to assess the immunosuppressive function of MDSCs, we employed ELISA kits (JONLNBIO, China) to quantify Arg1 and iNOS in the serum.

### 2.5 Correlation analysis

The correlations between the frequencies of PD-L1^+^MDSCs and TLR4^+^MDSCs with ALT and AST were analyzed in patients with brucellosis. This analysis aimed to elucidate the relationship between liver injury induced by brucellosis and the frequencies of MDSCs. Additionally, further correlation analyses were conducted to explore the relationship between PD-L1^+^MDSCs and TLR4^+^MDSCs with inflammation-related variables Arg1 and iNOS, thereby determining the association between MDSC subsets and inflammatory factors.

### 2.6 Construction of mouse model with *Brucella* infection

To establish the mouse infection model, 18 female BALB/c mice (6–8 weeks old, 15–20 g) were selected. The mice were randomized into two groups: the normal saline group (*n* = 6) received intragastric administration of 100 μL 0.9% normal saline, while the model group (*n* = 12) was intragastrically administered 100 μL of *Brucella melitensis* suspension (1 × 10^10^ CFU/mL). The model group was further subdivided into the acute brucellosis infection (ABI, 14 days) and the chronic brucellosis infection (CBI, 56 days). After the infection cycle ended, all the mice were raised separately and samples were collected regularly. The bacterial loads in the liver, spleen, and lung were quantified. The ZX-400 fully automatic colony counter was employed for colony enumeration and imaging, and the number of colony-forming units (CFU) per organ was subsequently calculated. This study has received approval from the Ethics Committee of the First Affiliated Hospital of Xinjiang Medical University, with the ethics number 20250424-100.

### 2.7 Histological analysis

Liver, spleen, and lung tissues of infected mice was processed for routine hematoxylin–eosin (HE) and immunohistochemical staining. Tissues were fixed in formalin and embedded in paraffin. HE staining was performed using HE staining kit (Solarbio, China) to systematically evaluate the pathological effects of *Brucella* infection. Immunohistochemical staining was employed to identify the localization of *Brucella* colonization in mouse organs. Immunohistochemical staining was performed by incubating the sections overnight at 4 °C with rabbit anti-*Brucella* (Bioss, China). Following rinsing, the sections were incubated with a appropriate secondary for 1 h at room temperature. Slices were imaged using a Nikon ECLIPSE Ni-U microscope.

### 2.8 Multiple immunofluorescence staining (mIF)

To further investigate the role and distribution of MDSCs during *Brucella* infection, we conducted mIF analysis of MDSCs. The primary antibodies, including mouse anti-CD11b (ABclonal, Chian), anti-Gr-1(ABclonal, Chian), and anti-Bru antibodies (Bioss, China), were incubated prior to the addition of the secondary antibody, which was a conventional goat anti-mouse antibody (Bioss, China). Add 1xDAPI (ZSGB-Bio, China) ensuring an even coverage of the tissue. Subsequently, seal the plate using a fluorescence quenching mounting medium. The fluorescence microscope revealed that CD11b emitted red fluorescence, Gr-1 emitted green fluorescence, *Brucella* emitted pink fluorescence, and DAPI emitted blue fluorescence.

### 2.9 Flow cytometric analysis of mice

Livers, lungs, and spleens were collected from mice to enumerate MDSCs by flow cytometry using antibodies specific for mouse MDSC markers. The tissues were homogenized to generate a single-cell suspension. The antibody panel for MDSC analysis was made up of anti-CD11b APC, anti-Gr-1 FITC, anti-TLR4 Bv421, and anti-PD-L1 PE and all from BD Biosciences. The antibodies were incubated in the dark for 20 min in preparation for machine detection. A BD FACS Canto Plus Flow Cytometer was used to acquire flow cytometric data. Data analysis was performed using FlowJo analysis software.

### 2.10 Statistical analysis

The statistical analysis of the experimental data in this study was conducted using GraphPad Prism 10 software. For quantitative data that follows a normal distribution, results are presented as mean ± standard deviation. For between-group comparisons, the independent samples *t*-test was applied to normally distributed variables, whereas the Wilcoxon rank-sum test was used for non-normally distributed variables. Differences across multiple groups were analyzed using one-way analysis of variance (ANOVA). The Spearman correlation coefficient was calculated to assess the linear relationship between variables, with a *p*-value of less than 0.05 signifying a statistically significant correlation among the variables.

## 3 Results

### 3.1 Clinical manifestations

This study included a total of 88 patients with ABI group with a mean age of 44.64 ± 10.51, comprising 63 males and 25 females. A total of 66 CBI patients group comprised 46 males and 20 females with a mean age of 43.45 ± 9.43 years. In total, there were 82 HC group included 62 males and 20 females, with a mean age of 42.42 ± 10.63 years. The age and gender were matched between cohorts, no significant differences in age and gender were observed. Among the 154 patients, 93 (60.39%) had a documented history of cattle and sheep breeding or direct contact with these animals. An additional 38 (24.68%) reported exposure to undercooked meat and dairy products. The remaining 23 patients (14.94%) had no identifiable epidemiological history associated with brucellosis acquisition. The laboratory and clinical indicators of brucellosis patients collected are presented in Table 1.

**Table 1:** Laboratory and clinical indexes of *brucellosis* patients.

Clinical characteristics	Acute stage (*n* = 88)	Chronic stage (*n* = 66)	*P*
WBC (×10^9^/L)	4.89 ± 2.19	5.85 ± 2.38	<0.001
HB (g/L)	115.72 ± 19.52	119.23 ± 20.36	<0.001
PLT (×10^9^/L)	181.01 ± 106.15	234.71 ± 99.32	<0.001
CR (umol/L)	58.04 ± 20.86	69.33 ± 39.27	0.043
CA (mmol/L)	2.12 ± 0.15	2.14 ± 0.13	0.387
ESR (mm/h)	32.88 ± 15.77	36.97 ± 13.90	0.126
CRP (mg/L)	36.46 ± 22.12	41.32 ± 27.99	0.237
PCT (ng/mL)	0.31 ± 0.65	0.18 ± 0.24	0.15
SAA (mg/mL)	115.61 ± 87.22	117.17 ± 107.12	0.256
Enlargement of liver, spleen	35(39.8)	55(22.7)	0.025
Arthralgia	37(42.0)	47(71.2)	<0.001

Noted Imaging examination: Enlargement of spleen is defined as a longitudinal diameter > 12 cm and thickness > 4 cm, while enlargement of liver is defined as a maximum oblique diameter of the right hepatic lobe > 14 cm and thickness of the left hepatic lobe > 6 cm.

### 3.2 Increased frequencies of TLR4^+^ MDSCs, and PD-L1^+^ MDSCs in patients with acute and chronic brucellosis

**Figure 1:**
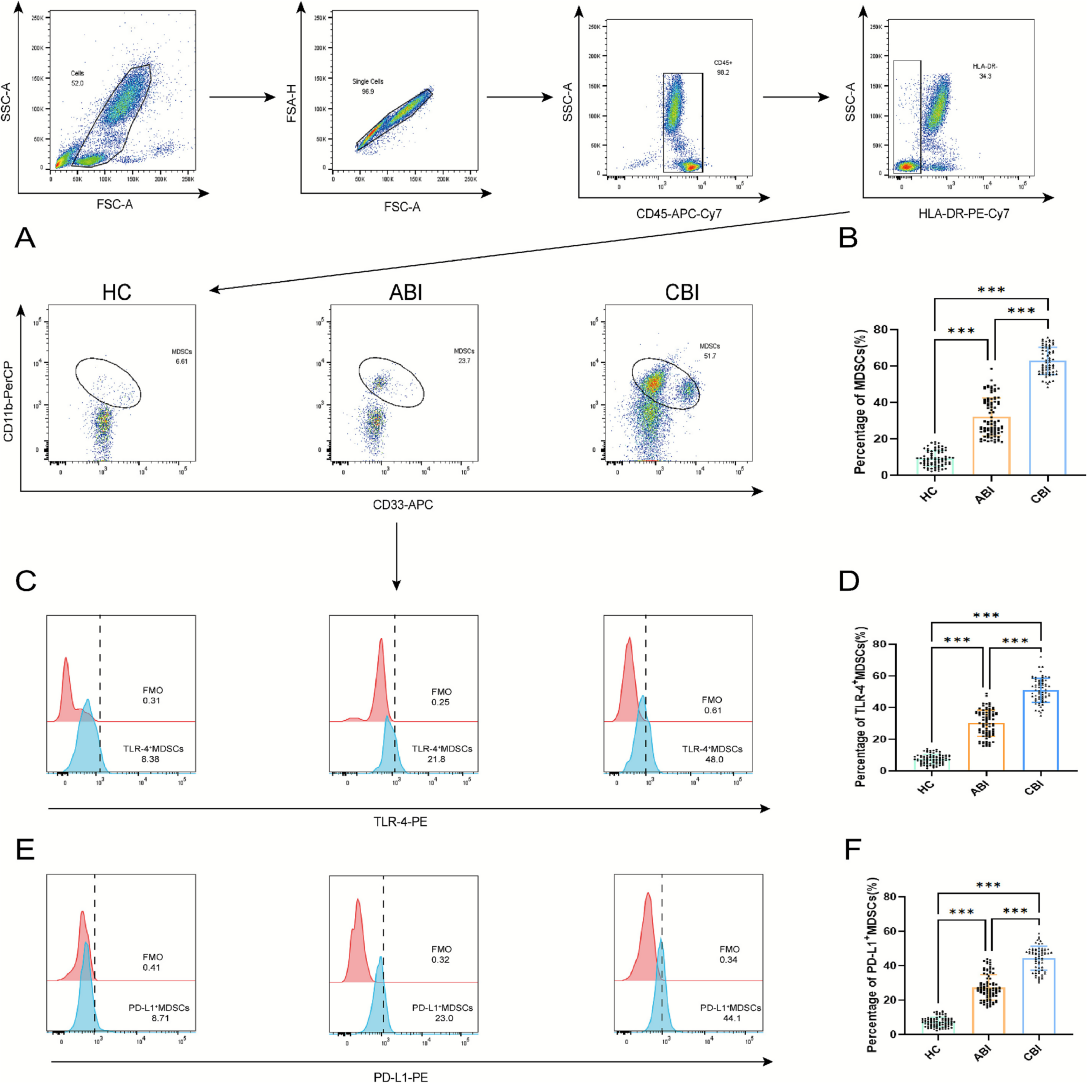
Illustrates the gating strategy for MDSCs and their subgroups in HC group, ABI group, and CBI group as analyzed by flow cytometry. **(A)** MDSCs flow chart; **(B)** percentage and difference analysis of MDSCs; **(C)** TLR4^+^MDSCs flow chart; **(D)** percentage and difference analysis of TLR4^+^MDSCs; **(E)** PD-L1^+^MDSCs flow chart; **(F)** percentage and difference analysis of PD-L1^+^MDSCs (the data are presented as the mean ± SD. ABI, *n* = 88; CBI, *n* = 66; HC, *n* = 82. ^***^p < 0.001).

In this study, we detected peripheral blood MDSCs, included 82 HC subjects, 88 ABI patients, 66 CBI patients. Compared to the HC group, the frequencies of MDSCs significantly increased in both the ABI and CBI groups, particularly in the CBI group (*p* < 0.001). Additionally, TLR4^+^MDSCs exhibited a significant increase in the infected groups, especially in the CBI group (*p* < 0.001). The results of PD-L1^+^MDSCs showed that the CBI group was also significantly higher than the ABI and control groups, as illustrated in Figure 1.

### 3.3 Association of TLR4^+^MDSCs and PD-L1^+^MDSCs with liver damage and immune responses in *Brucella* infection

**Figure 2:**
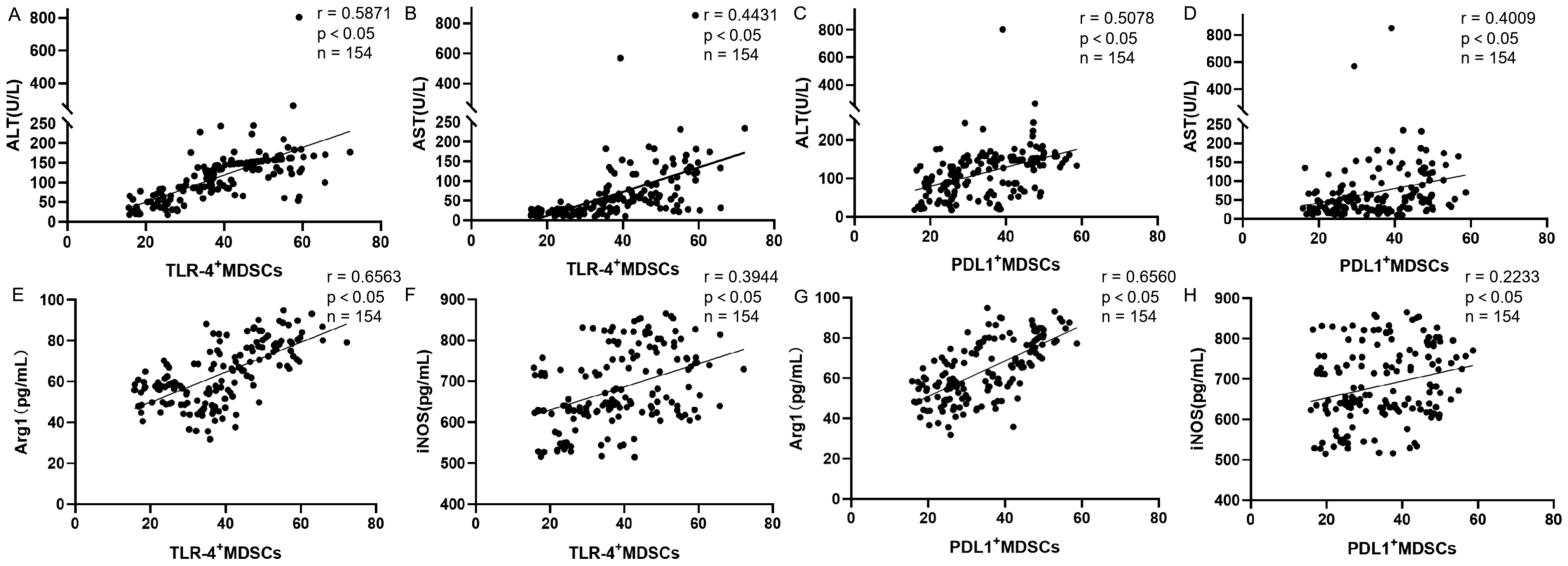
Correlation analysis between TLR4+MDSCs level and PD-L1+MDSCs levels and serum ALT, AST, Arg1, and INOS in patients with brucellosis (*n* = 154; R, Spearman correlation coefficient). **(A)** Correlation between the proportion of TLR-4^+^MDSCs and serum ALT levels. **(B)** Correlation between the proportion of TLR-4^+^MDSCs and serum AST levels. **(C)** Correlation between the proportion of PD-L1^+^MDSCs and serum ALT levels. **(D)** Correlation between the proportion of PD-L1^+^MDSCs and serum AST levels. **(E)** Correlation between the proportion of TLR-4^+^MDSCs and serum Arg1 levels. **(F)** Correlation between the proportion of TLR-4^+^MDSCs and serum iNOS levels. **(G)** Correlation between the proportion of PD-L1^+^MDSCs and serum Arg1 levels. **(H)** Correlation between the proportion of PD-L1^+^MDSCs and serum iNOS levels.

In this study, we evaluated the levels of ALT, AST, Arg1, and iNOS in 154 patients diagnosed with *Brucella*. We assessed the correlation between these four parameters and TLR4^+^MDSCs and PD-L1^+^MDSCs. Our findings indicate that TLR4^+^MDSCs levels and PD-L1^+^MDSCs levels are positively correlated with both AST and ALT. Furthermore, an increase in TLR4^+^MDSCs and PD-L1^+^MDSCs is associated with exacerbated liver damage in patients. Additionally, serum levels of Age1 and INOS also show a positive correlation with TLR4^+^MDSCs levels and PD-L1^+^MDSCs levels (Figure 2).

### 3.4 Bacterial load increase in *Brucella*-infected mice

**Figure 3:**
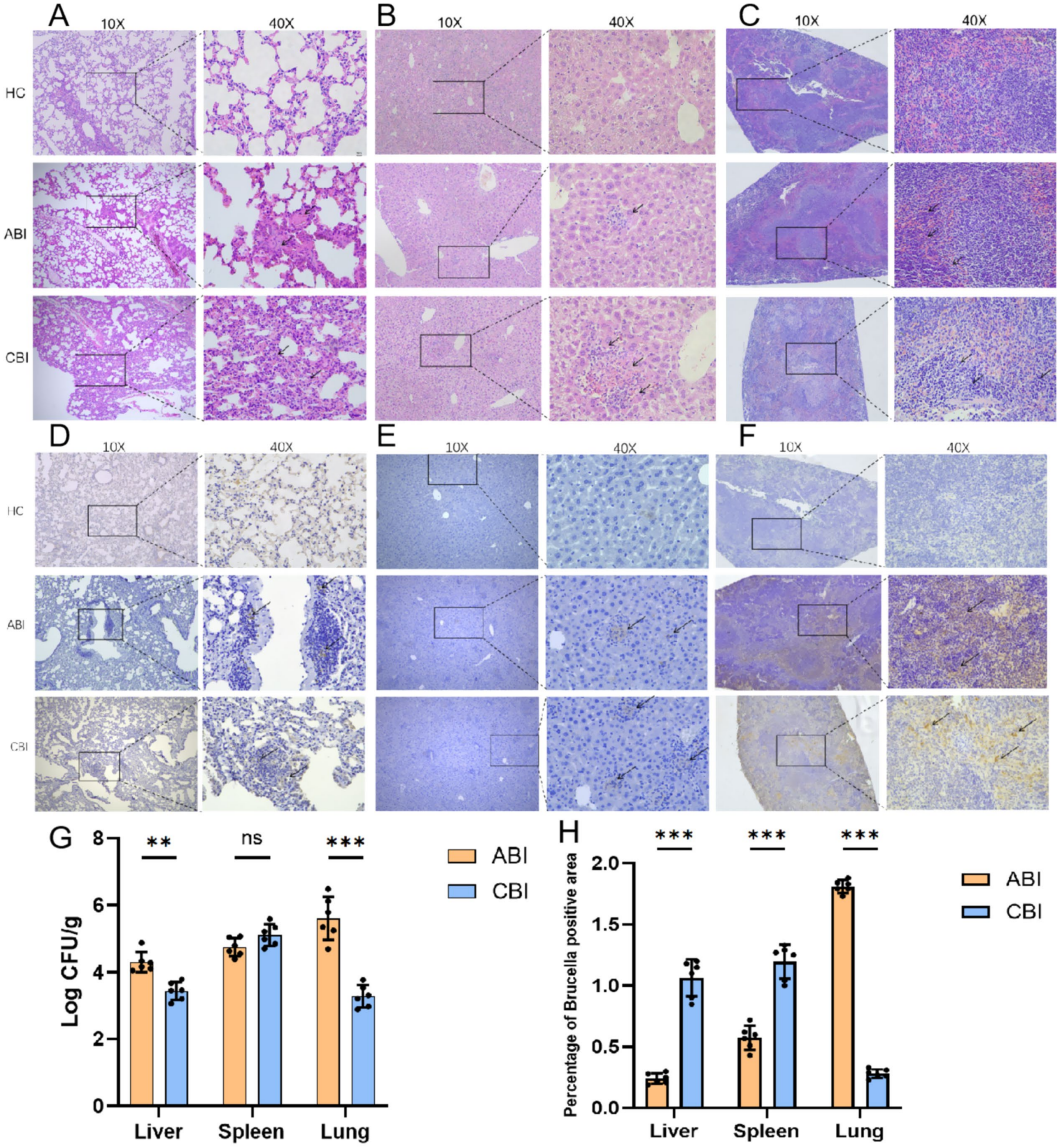
Histopathological staining of mouse organs, alongside bacterial colonization assessments. **(A)** Lung HE staining; **(B)** Liver HE staining; **(C)** Spleen HE staining. **(D)** Lung IHC staining; **(E)** Liver IHC staining; **(F)** Spleen IHC staining; **(G)** Statistical diagram of colony count in various organs of mice; **(H)** Percentage of *Brucella* positive areas. (ABI, *n* = 6; CBI, *n* = 6. ^**^p < 0.01, ^***^p < 0.001, ns, no significance).

To quantitatively assess the extent of *Brucella* infection and the associated modeling, bacterial cultures were performed on the liver, spleen, and lungs of mice. The experimental results indicated that the bacterial load in the liver (*p* < 0.01) and lungs (*p* < 0.001) significantly increased during the ABI stage, whereas the bacterial load in the spleen remained elevated in both the acute and chronic stages, with no significant differences observed (Figure 3G).

### 3.5 Histopathological changes in liver, lung, and spleen of mice infected with *Brucella*

HE staining indicated that the liver, lung, and spleen structures of mice in the control group were normal, with no infiltration of inflammatory cells observed. In ABI group, lung (Figure 3A) and the liver (Figure 3B) of the mice exhibited pronounced signs of inflammation, while the spleen (Figure 3C) showed partial dilation of the marginal zone and slight widening of the splenic trabeculae. The boundary between the red and white pulp began to blur. The inflammatory response in the CBI group was more pronounced, with extensive infiltration of inflammatory cells in the liver, an increase in inflammatory exudate in the lungs, and localized thickening of the alveolar walls. The spleen demonstrated the most significant destruction, characterized by necrotic areas and suspected replacement with fibrous connective tissue. The marginal area was extensively dilated, the trabeculae of the spleen were widened and thickened, and the boundary between the red and white marrow was nearly indistinguishable. Through immunohistochemical analysis, we observed that, compared to the HC group, mice with ABI exhibited increased *Brucella* colonization in the lungs (Figure 3D), liver (Figure 3E), and spleen (Figure 3F). However, during the chronic infection stage, colonization in the liver and spleen further increased, while *Brucella* antigen levels in the lungs decreased compared to the acute stage (Figure 3H) (all *p* < 0.001).

### 3.6 Enrichment of MDSCs in mice with chronic brucellosis

**Figure 4:**
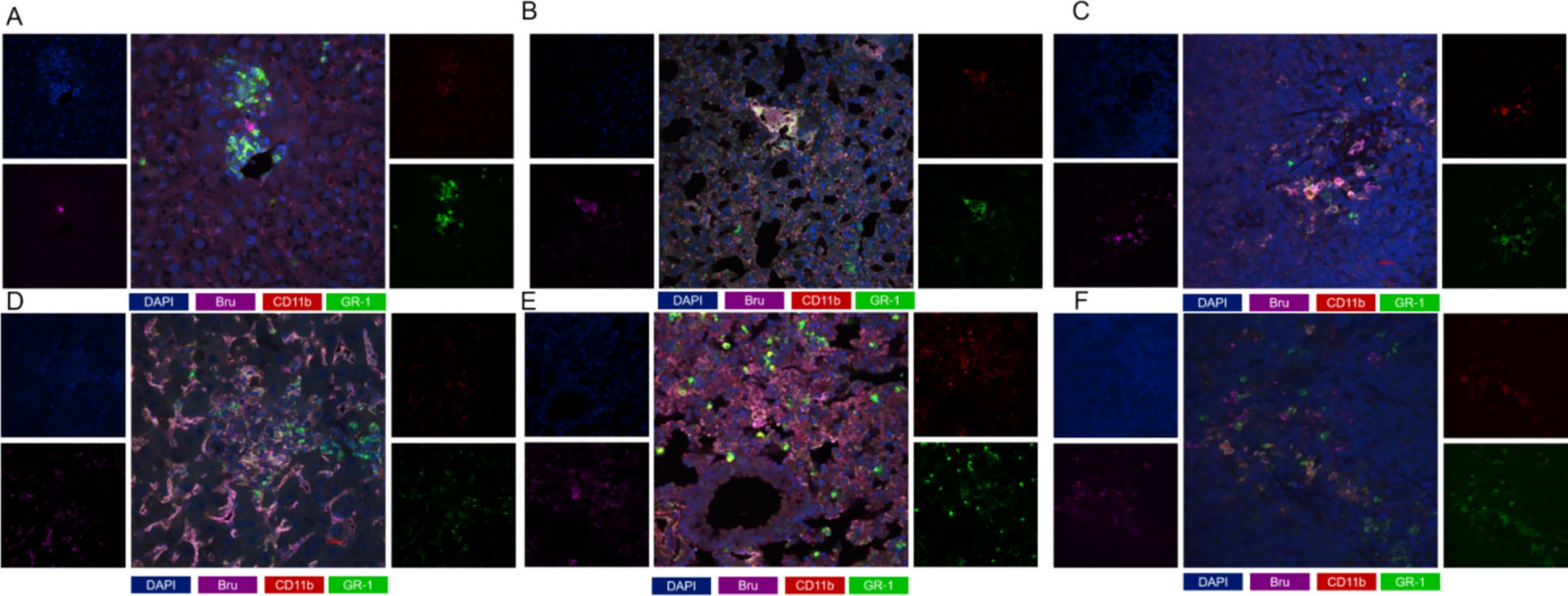
mIF staining results of mouse lungs, livers and spleens. **(A)** mIF staining in acute liver; **(B)** mIF staining of lung in acute stage; **(C)** mIF staining in acute spleen; **(D)** mIF staining of chronic liver; **(E)** mIF staining of lung in chronic stage; **(F)** mIF staining of spleen in chronic stage. (blue: DAPI; Purple: Bru; Red: CD11b; Green: GR-1).

Co-localization analysis of CD11b and Gr-1 revealed limited MDSC proliferation across multiple organ tissues in ABI group, whereas significant proliferation and activation were observed in the CBI group. With the progression of brucellosis to chronicity, MDSC levels in each organ gradually increased, suggesting a strong correlation between enhanced MDSC activation and brucellosis progression. Notably, MDSC infiltration in the liver (Figures 4A, D), lung (Figures 4B, E), and spleen (Figures 4C, F) closely matched the antigen localization of *Brucella*, indicating that *Brucella* infection may recruit MDSCs to infection sites to participate in local immune regulation.

### 3.7 Enhanced frequencies of TLR4^+^ MDSCs and PD-L1^+^ MDSCs in mice with brucellosis

**Figure 5:**
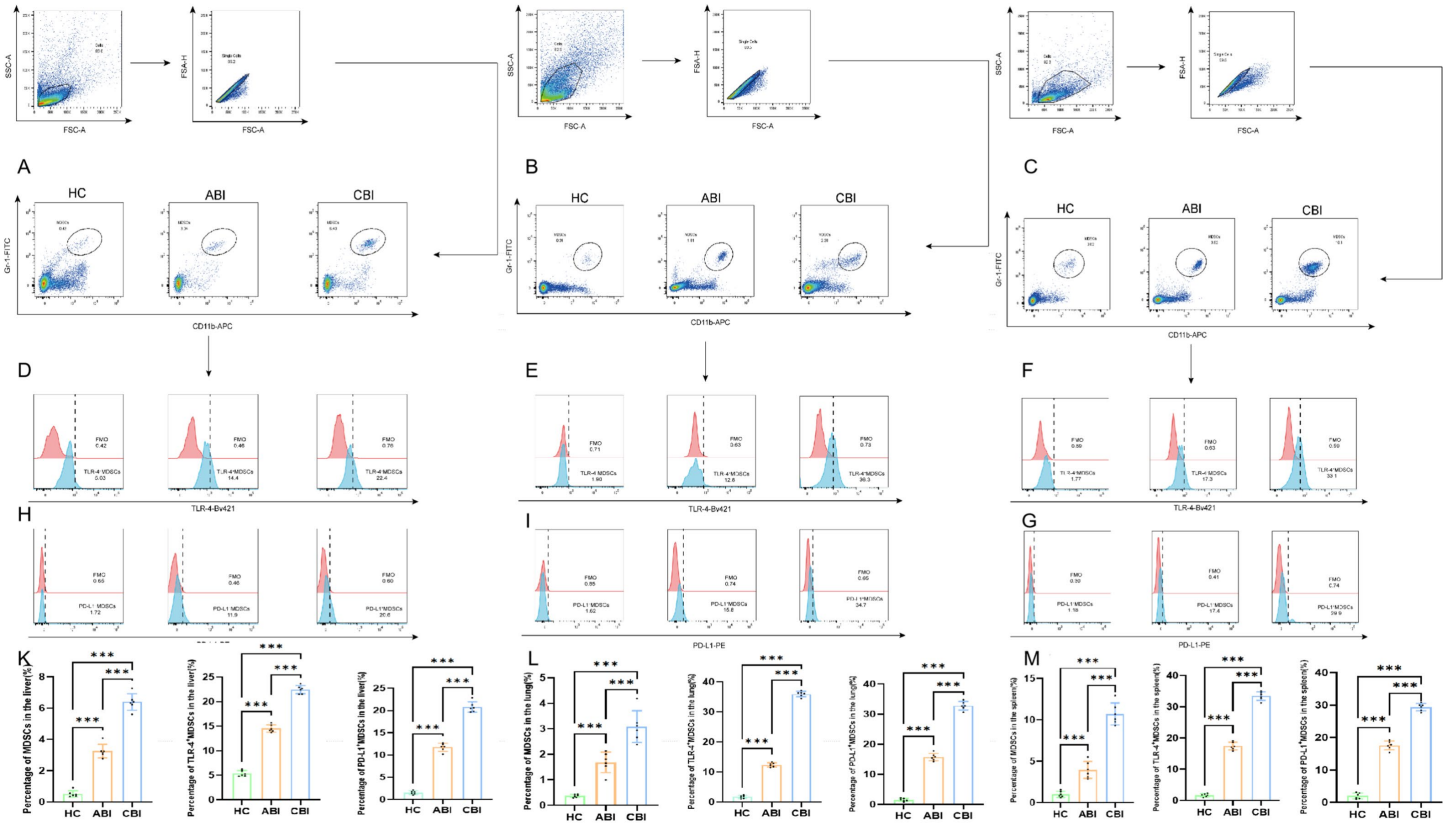
**(A)** Flow cytometry results of liver MDSC in mice; **(B)** low cytometry results of lung MDSC in mice; **(C)** low cytometry results of spleen MDSC in mice; **(D)** low cytometry results of liver TLR4^+^MDSCs in mice; **(E)** low cytometry results of lung TLR4^+^MDSCs in mice; **(F)** low cytometry results of spleen TLR4^+^MDSCs in mice; **(H)** low cytometry results of liver PD-L1^+^MDSCs in mice; **(I)** low cytometry results of lung PD-L1^+^MDSCs in mice; **(G)** low cytometry results of spleen PD-L1^+^MDSCs in mice; **(K)** percentage and difference analysis of liver in mice; **(L)** percentage and difference analysis of lung in mice; **(M)** percentage and difference analysis of spleen in mice. (ABI, *n* = 6; CBI, *n* = 6. ^***^p < 0.001).

Flow cytometric analysis of mouse liver (Figure 5A), lung (Figure 5B), and spleen (Figure 5C) tissues revealed that MDSC frequencies in CBI group were significantly higher than those in ABI group and healthy controls (*p* < 0.001). Similarly, TLR4^+^ MDSCs and PD-L1^+^ MDSCs were markedly elevated in chronic mice compared to acute mice and healthy controls (Figures 5D-M) (*p* < 0.001). Collectively, significant accumulation of TLR4^+^ MDSCs and PD-L1^+^ MDSCs in chronic brucellosis suggests these subsets may drive disease chronicity and represent potential therapeutic targets.

## 4 Discussion

Brucellosis is a zoonotic disease. The symptoms of this infection are non-specific and persistent, leading to chronic infection within the body. It is commonly associated with the consumption of infected dairy products and has resulted in significant economic losses and personal injuries worldwide (Xie et al., 2025). Due to the non-specific nature of its clinical symptoms, brucellosis is often misdiagnosed, which can result in a progression to the chronic stage. More importantly, if appropriate treatment is not administered during the acute stage, various complications may arise. All brucellosis patients enrolled in this study were confirmed to be infected with *Brucella melitensis*, which is the predominant pathogenic species of brucellosis in the Xinjiang region (Zhang et al., 2015; Yang et al., 2024). The diagnosis of brucellosis was based on typical clinical features and positive Rose Bengal Precipitation Test (RBPT) and/or Serum tube Agglutination Test (SAT) (≥1:100) And/or isolate Brucella from blood, other tissues or body fluids, and combine it with epidemiological history.

Among 154 patients diagnosed with brucellosis, 21.43% exhibited abnormalities in the liver and spleen, 18.83% presented with lumbar spine infections, and 17.63% experienced knee joint infections. These proportions are consistent with most previous reports (Zheng et al., 2019). Feng et al.’s study indicates that among patients with brucellosis complicated by additional conditions, 41.46% exhibit lumbar spine lesions, while 7.01% present with splenomegaly and lymphadenopathy (Qiangsheng et al., 2023). The infection poses a significant threat to the liver, spleen, and skeletal system of affected patients. However, to date, the pathogenesis of brucellosis remains inadequately understood.

MDSCs exhibit potent immunosuppressive activity by inhibiting effective T-cell immune responses, thereby facilitating pathogen persistence and the development of chronic infections (Arshad et al., 2024). MDSCs also serve as critical drivers of tumor progression, exerting significant impacts on tumor angiogenesis, metastasis, and multidrug resistance (MDR)(Lasser et al., 2024; Dhar et al., 2023). In the context of gastric cancer research, MDSCs have been identified as novel targets for early diagnosis, intervention, and precision treatment of this malignancy (Li et al., 2024). High-virulence *Klebsiella pneumoniae* (hvKp) infection drives MDSC infiltration into lung tissues, where MDSCs inhibit T-cell proliferation and induce apoptosis via indoleamine 2,3-dioxygenase 1 (IDO1)-mediated tryptophan metabolism, leading to lymphopenia and persistent infection (Xu et al., 2025). Furthermore, investigations have demonstrated that MDSCs also exert immunomodulatory effects in other infectious diseases, such as infections caused by *Staphylococcus aureus*, *hepatitis B virus*, and *Candida albicans* (Sanchez-Pino et al., 2021; Bruger et al., 2019). MDSCs promote immunosuppression through the upregulation of immunosuppressive mediators, including Arg1 and iNOS, which leads to antigen-presenting cell (APC) dysfunction, reduced activation of T effector cells (Teff), and enhanced recruitment, survival, and functionality of regulatory T cells (Tregs)(Weber et al., 2021; Saleh et al., 2020).

In this study, we analyzed peripheral blood MDSCs in healthy controls and patients with acute and chronic brucellosis using flow cytometry. We evaluated the expression of TLR4 and PD-L1 on MDSCs. The results indicated that both the percentage and absolute number of MDSCs in patients during the chronic phase were significantly higher than those in the acute phase, suggesting that MDSCs may play a more critical role in chronic brucellosis. Furthermore, the numbers of TLR4^+^MDSCs and PD-L1^+^MDSCs in chronic phase patients were also elevated compared to those in acute phase patients. TLR4, a pattern recognition receptor, is pivotal in innate immune responses, as its activation can promote inflammatory responses and enhance the activation of immune cells (Zhang et al., 2020; Strobl et al., 2022). However, persistent activation of TLR4 may lead to immune system fatigue, thus facilitating the accumulation of MDSCs and resulting in immunosuppression (Sun et al., 2018; Shime et al., 2017). PD-L1, an essential immune checkpoint molecule, exhibits high expression on MDSCs, potentially augmenting their immunosuppressive function and aiding *Brucella* in evading immune surveillance. Therefore, the elevated expression of TLR4 and PD-L1 on MDSCs may represent a significant mechanism underlying the immunosuppressive status observed in patients during the chronic phase.

MDSCs exert a role in liver injury: they can either exacerbate liver damage and drive fibrogenesis via orchestrating inflammatory responses and immune suppression, or confer hepatoprotection by constraining excessive inflammatory cascades in specific pathological settings (Lu et al., 2025; Luo et al., 2022). ALT and AST are widely utilized in clinical diagnostics to evaluate liver health and tissue damage (Choi et al., 2024). The correlation analysis between the levels of ALT and AST and the cell counts of TLR4^+^MDSCs and PD-L1^+^MDSCs indicates that following *Brucella* infection, an increase in the numbers of TLR4^+^MDSCs and PD-L1^+^MDSCs corresponds with elevated ALT and AST levels, leading to liver damage. This suggests that these subsets of MDSCs may play a critical role in liver injury associated with *Brucella* infection. Furthermore, this study reveals that the levels of inflammatory factors, such as Arg1 and iNOS, are directly proportional to the fluctuations in the numbers of TLR4^+^MDSCs and PD-L1^+^MDSCs. During the course of infection, these cell subsets are rapidly activated and express relevant cytokine, thereby exacerbating *Brucella* infection and liver injury.

Through the determination of organ bacterial load, as well as HE and IHC staining in a *Brucella* mouse model, we systematically evaluated the pathological changes in the liver, lung, and spleen tissues, along with the colonization of *Brucella* in the infected mice. In the chronic stage of the infection, the inflammatory response in the organs became more pronounced, indicating severe organ involvement. Results from the mIF experiment revealed a significant increase in MDSC cells within the liver, spleen, and lung tissues of the infected mice. Furthermore, with the progression of the infection, both the proliferation and activation levels of MDSCs gradually increased. This finding suggests that MDSCs may play a crucial role in the progression of *Brucella* infection, with their heightened activation closely associated with the chronicity of the disease. The flow cytometry results of mouse cells indicated that the frequencies of TLR4^+^MDSCs and PD-L1^+^MDSCs during the chronic phase were significantly higher than those observed in both the acute phase and the healthy control group. Although this suggests that TLR4^+^MDSCs and PD-L1^+^MDSCs may represent key cellular subsets in chronic brucellosis infection, playing a crucial role in regulating immune responses and promoting the persistence of the infection, this study has several limitations. It should be noted that the increased frequency of TLR4^+^ and PD-L1^+^ MDSCs along with disease chronicity may not be solely driven by disease progression per se, but also modulated by multiple confounding factors including systemic inflammatory status, tissue bacterial burden, host immune baseline heterogeneity, and potentially therapeutic interventions.

## 5 Conclusion

In summary, this research underscore the significant roles of the two functional subsets, TLR4^+^and PD-L1^+^MDSCs, in chronic brucellosis, thereby offering a novel perspective on the disease’s chronicity. These subsets may serve as potential biomarkers for evaluating the severity and progression of the disease. Furthermore, targeting the immunosuppressive properties of MDSC subsets may dramatically enhance the host’s antimicrobial immune responses, presenting a novel therapeutic avenue for brucellosis treatment. Future research can further explore the specific mechanism of action of MDSCs in *Brucella* immune escape and chronic infection.

## Data Availability

The original contributions presented in the study are included in the article/supplementary material, further inquiries can be directed to the corresponding authors.
